# Supplementary material for: Preoperative Typing of Thyroid and Parathyroid Tumors with a Combined Molecular Classifier
Source: Cancers (Basel). 2021 Jan 11;13(2):237. doi: 10.3390/cancers13020237 (PMC7827881; doi:10.3390/cancers13020237)
Supplement: Supplementary file 1 [file cancers-13-00237-s001.pdf]

Supplementary Materials

Preoperative Typing of Thyroid and Parathyroid Tumors with a Combined Molecular Classifier

Titov S.E. et al.

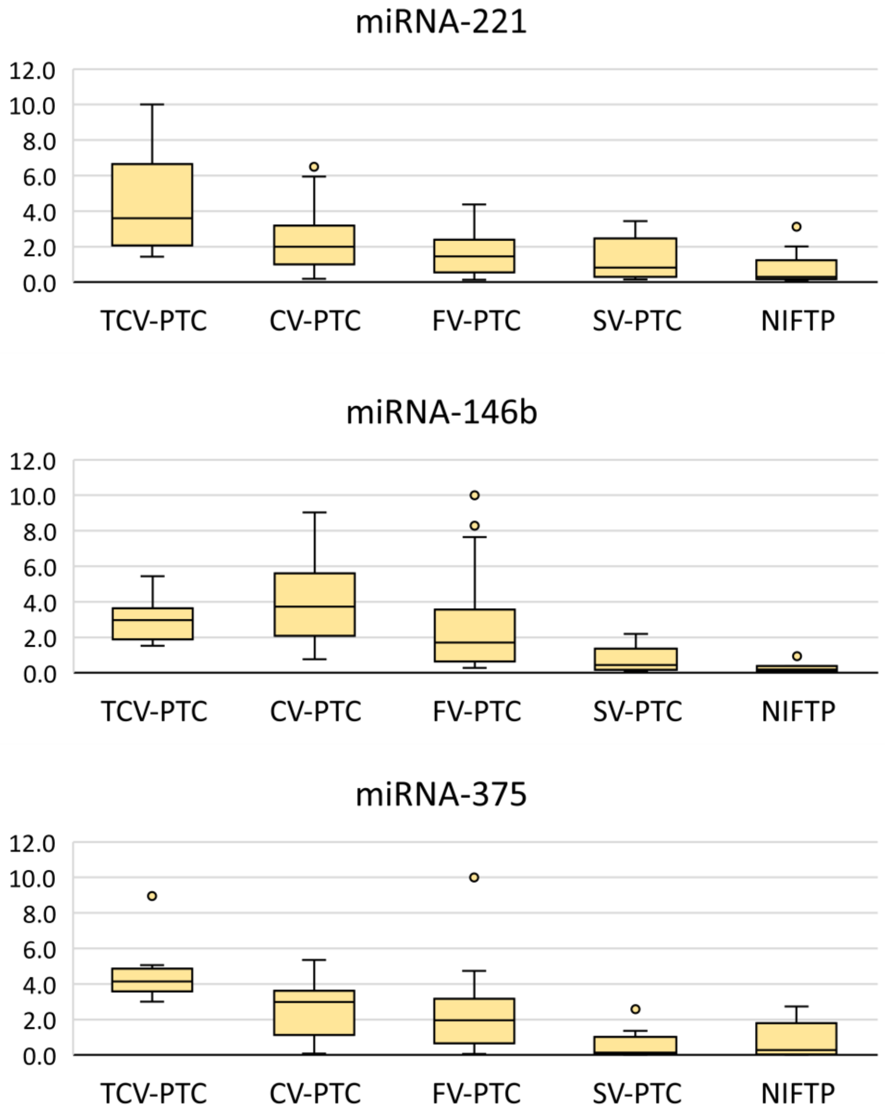

Figure S1. Relative expression levels of three miRNAs in different subtypes of PTC and NIFTP. The data are normalized; the figure shows the median value, upper and lower quartiles, a nonoutlier range, and outliers (circles). TCV-PTC, tall-cell variant of PTC; CV-PTC, classic variant of PTC; FV-PTC, follicular variant of PTC; SV-PTC, solid variant of PTC; NIFTP, noninvasive follicular thyroid neoplasm with papillarylike nuclear features.

Table S1. P values for pairwise comparisons of miRNA-146b, -221 and -375 expression between various types of PTC and NIFTP

		CV-PTC	FV-PTC	SV-PTC	NIFTP
TCV-PTC	miR-146b	0.446305	0.152607	**0.001764**	**0.000449**
	miR-221	**0.025011**	**0.004550**	**0.006099**	**0.002174**
	miR-375	**0.005001**	**0.001548**	**0.000636**	**0.000449**
CV-PTC	miR-146b		**0.037705**	**0.000157**	**0.000011**
	miR-221		0.106038	0.110335	**0.002658**
	miR-375		0.230741	**0.000854**	**0.000942**
FV-PTC	miR-146b			**0.013939**	**0.000052**
	miR-221			0.420757	**0.021616**
	miR-221			**0.005001**	**0.006065**
SV-PTC	miR-146b				0.111348
	miR-221				0.205668
	miR-375				0.902523

Significant ($p < 0.05$) differences are highlighted in bold.

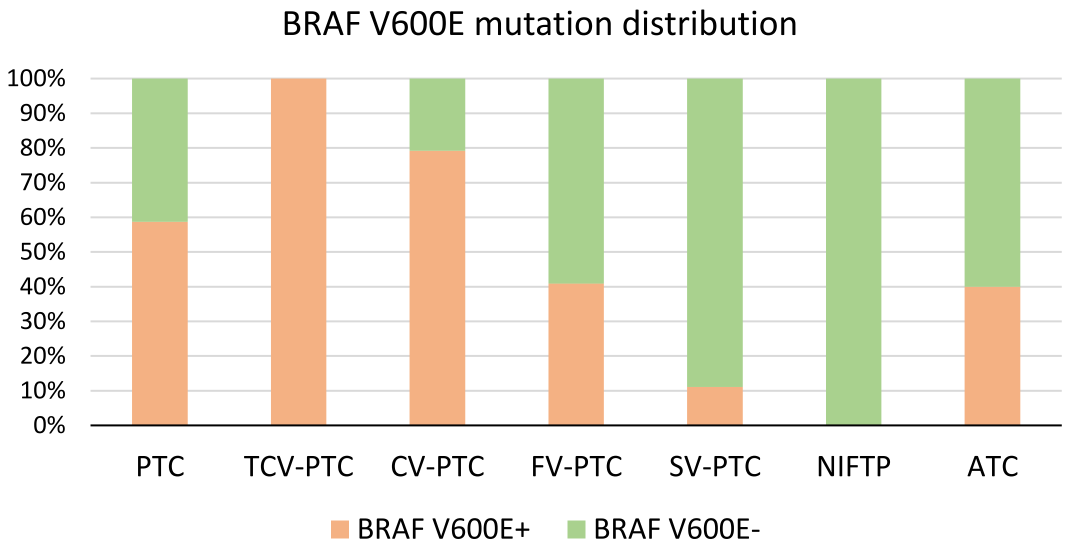

Figure S2. Prevalence of the *BRAF*^{V600E} mutation in ATC, NIFTP, and different subtypes of PTC. PTC, all variants of papillary thyroid carcinoma; TCV-PTC, tall-cell variant of PTC; CV-PTC, classic variant of PTC; FV-PTC, follicular variant of PTC; SV-PTC, solid variant of PTC; NIFTP, noninvasive follicular thyroid neoplasm with papillarylike nuclear features; ATC, anaplastic thyroid carcinoma.

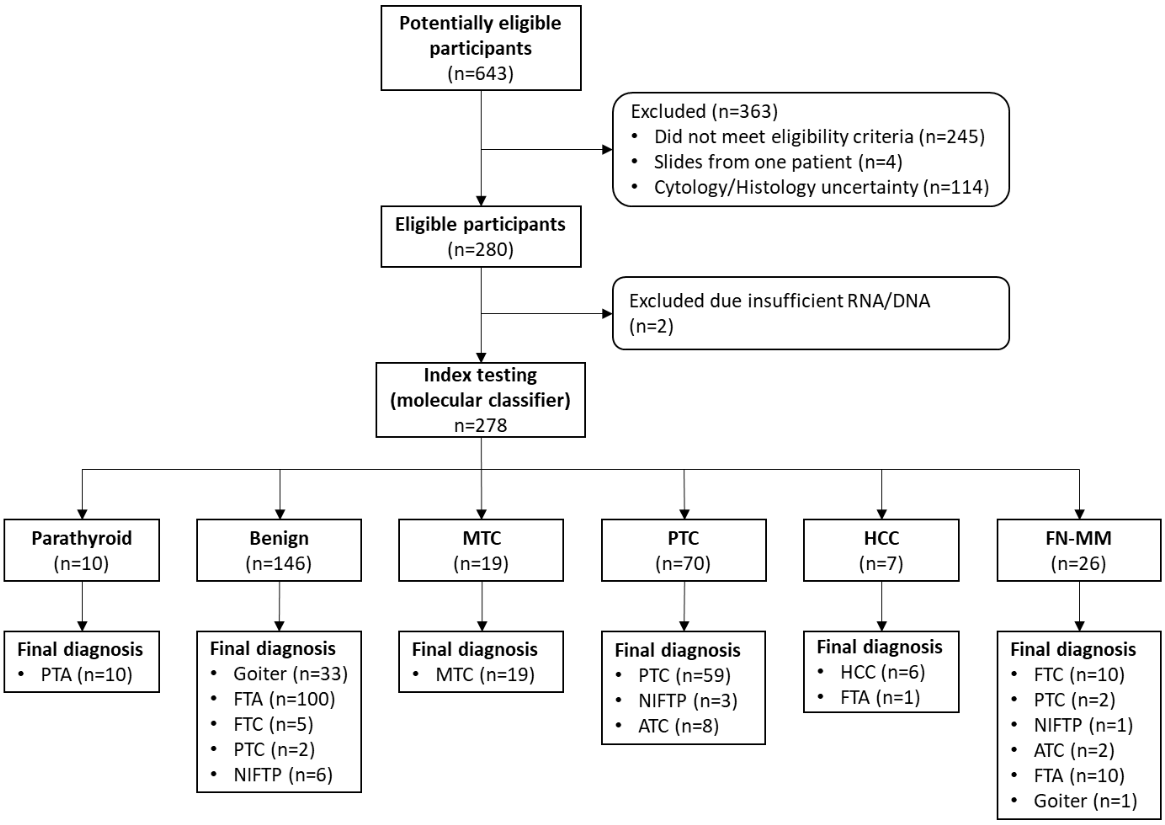

Figure S3. A Standards for Reporting of Diagnostic Accuracy Studies diagram of sample flow through this study.

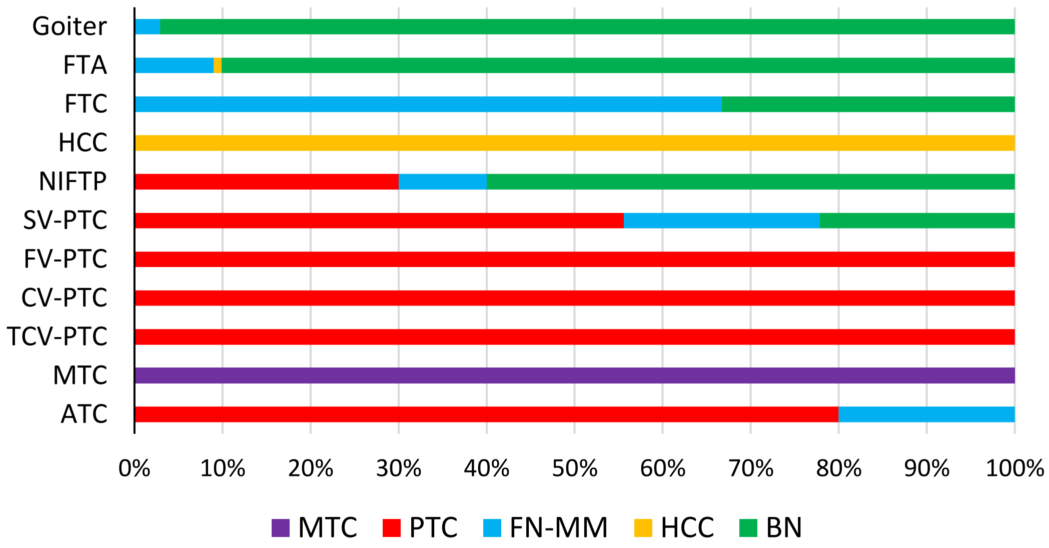

Figure S4. Proportions of samples belonging to different classes according to the molecular classifier but belonging to the same class according to a pathology report (i.e., histological examination; indicated on the left). ATC, anaplastic thyroid carcinoma; BN, benign nodule; CV-PTC, classic variant of PTC; FN-MM, follicular neoplasm with markers of malignancy; FTA, follicular thyroid adenoma; FTC, follicular thyroid carcinoma; FV-PTC, follicular variant of PTC; HCC, Hürthle cell carcinoma; MTC, medullary thyroid carcinoma; NIFTP, noninvasive follicular thyroid neoplasm with papillarylike nuclear features; SV-PTC, solid variant of PTC; TCV-PTC, tall-cell variant of PTC.

Table S2. The patients' characteristics.

	No. of samples	Sex, No. (%) of female patients	Age of patients, mean (range)	Size on ultrasonography, mean (range), cm
FTA	111	84 (75.7)	50 (18-73)	2.8 (0.5-13.0)
FTC	21	15 (71.4)	46 (28-72)	2.7 (1.1-5.0)
FV-PTC	22	19 (86.3)	53 (18-71)	1.9 (1.0-5.7)
TCV-PTC	8	5 (62.5)	53 (41-69)	1.7 (0.5-3.0)
SV-PTC	9	7 (77.7)	37 (18-57)	1.6 (0.5-2.5)
CV-PTC	24	19 (79.2)	44 (20-68)	1.9 (0.5-3.7)
NIFTP	10	7 (70)	50 (29-70)	2.7 (1.4-7.0)
MTC	19	16 (84.2)	54 (23-80)	1.5 (0.5-5.5)
ATC	10	8 (80)	70 (50-85)	7.1 (1.0-12.0)
PTA	10	10 (100)	51 (36-66)	1.4 (1.0-2.0)
Goiter	34	28 (82.3)	52 (23-72)	3.4 (0.5-10.0)
**All samples**	**278**	**218 (78.4)**	**51 (18-85)**	**2.5 (0.5-13.0)**

© 2020 by the authors. Licensee MDPI, Basel, Switzerland. This article is an open access article distributed under the terms and conditions of the Creative Commons Attribution (CC BY) license (http://creativecommons.org/licenses/by/4.0/).
